# Engineering the Charge Transfer in all 2D Graphene-Nanoplatelets Heterostructure Photodetectors

**DOI:** 10.1038/srep24909

**Published:** 2016-05-04

**Authors:** A. Robin, E. Lhuillier, X. Z. Xu, S. Ithurria, H. Aubin, A. Ouerghi, B. Dubertret

**Affiliations:** 1 Laboratoire de Physique et d’Étude des Matériaux, PSL Research University, CNRS UMR 8213, Sorbonne Universités UPMC Univ Paris 06, ESPCI ParisTech, 10 rue Vauquelin, 75005 Paris, France; 2 Nexdot, 10 rue Vauquelin, 75005 Paris, France; 3 UPMC –CNRS UMR 7588, Institut des Nano-Sciences de Paris (INSP), 4 place Jussieu, 75005 Paris, France; 4 Laboratoire de Photonique et Nanostructures, CNRS, Route de Nozay, 91460, Marcoussis, France

## Abstract

Two dimensional layered (*i.e*. van der Waals) heterostructures open up great prospects, especially in photodetector applications. In this context, the control of the charge transfer between the constituting layers is of crucial importance. Compared to bulk or 0D system, 2D materials are characterized by a large exciton binding energy (0.1–1 eV) which considerably affects the magnitude of the charge transfer. Here we investigate a model system made from colloidal 2D CdSe nanoplatelets and epitaxial graphene in a phototransistor configuration. We demonstrate that using a heterostructured layered material, we can tune the magnitude and the direction (*i.e*. electron or hole) of the charge transfer. We further evidence that graphene functionalization by nanocrystals only leads to a limited change in the magnitude of the 1/f noise. These results draw some new directions to design van der Waals heterostructures with enhanced optoelectronic properties.

Colloidal quantum dots (CQD) are attracting growing interest since their recent use in displays as narrow luminescence fluorophores. One of the promising next steps is their integration in optoelectronic devices such as photodetectors or photovoltaic cells^1^,^2^,^3^. In a CQD array, the transport occurs through a hopping process which tends to limit the mobility below ≈1 cm^2^.V^−1^.s^−1 ^4,5. As a consequence, the carrier diffusion length remains limited: 100–200 nm^6^, which is a decade below the absorption depth of the nanocrystals thin film. Various strategies have been explored to overcome the transport bottleneck, such as building phototransistors^7^ to fill majority carrier traps or reducing the electrode spacing using a nanotrench geometry to get rid of hopping transport^8^,^9^.

As an alternative, van der Waals heterostructures can be used to decouple the photogeneration from the transport. Such devices can be built by coupling CQD with efficient transport layer such as graphene^10^,^11^,^12^. Hybrid graphene/CQDs photoconductors with CQDs of various composition including PbS^13^,^14^, ZnO^15^, CdSe^16^ and CdS^17^ have been realized. A photoconductive gain of 10^8^ and responsivity of 10^7^ A.W^−1^ can be achieved^13^ at very low irradiance (10^−14^ W.cm^−2^), thanks to the high mobility of the graphene channel and the long carrier lifetime of the photogenerated carriers in the CQD layer.

In spite of all these progresses, the performances of photoconductors are limited by several parameters. Some of them have already been addressed^18^, such as the carrier mobility of the CQD film, CQD surface chemistry^19^, and the doping of the CQD layer^20^. But other parameters, including the effect of the CQD environment (the gas for example), the number of charges that can be trapped on the CQDs, the lifetime of these trapped charges, the effect of heterostructures on charge separation, are still largely undocumented. In particular, purely 2D heterostructures strongly differ from nanocrystal-graphene hybrid systems due to their large exciton binding energy which potentially prevents charge transfer to other layers^21^,^22^,^23^,^24^. In this paper, we highlight and propose some strategies to reduce the impact of this large binding energy by forming in-plane heterostructures. To do so and rather than using conventional Transition Metal DiChalcogenide (TMDC) material such as MoS_2_ or WS_2_, we use colloidally grown CdSe nanoplatelets (NPL). In addition to their 2D character^25^,^26^, the NPL offer the possibility to be grown under a heterostructured core/shell and core-crown aspect to tune their carrier localization^27^,^28^. These NPL-graphene hybrid systems are studied in an electrolyte gated phototransistor configuration to finely probe the carrier density within the graphene and evidence the charge transfer from the semiconductor layer. Moreover, we also studied how the dynamic of transport is affected and show that the 1/f noise intensity in the graphene layer is only moderately affected by the graphene functionalization by nanocrystals.

## All 2D nanoplatelets-graphene heterostructure in an electrolytic gating configuration

**Figure 1:**
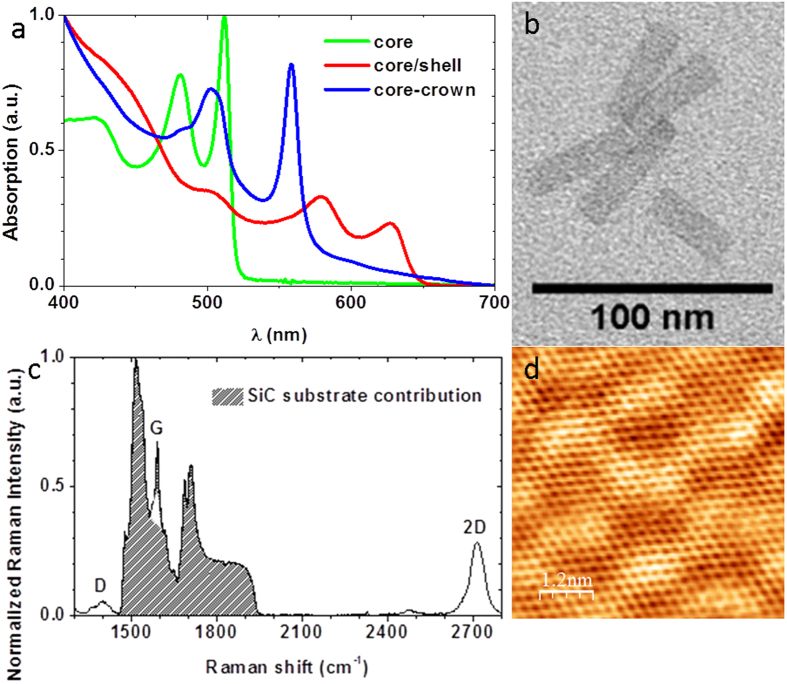
(**a**) Absorbance spectra of a suspension of CdSe core (green line), CdSe/CdS core/shell (red line) and CdSe-CdTe core-crown (blue line) nanoplatelets. (**b**) TEM picture of CdSe core nanoplatelets. (**c**) Raman spectra of a hydrogenated graphene layer on the Si-face of 4H-SiC. (**d**) STM image (−50 mV, 0.1 nA) of such a graphene sample.

Colloidal metal-chalcogenides nanoplatelets (NPL) such as CdSe offer excellent optical properties originating from the control of their thickness at the atomic level^29^,^30^. As synthetized^26^, they exhibit sharp excitonic features (see Fig. 1a – green curve and b) which make them appealing candidates for photodetectors with well-defined cut-off wavelength. The initial long capping ligands are exchanged for atomically short sulfide ligands^31^ to reduce the interlayer spacing and favour the charge transfer (see Supplementary Information S1 for additional details on synthesis and ligand exchange). In the meanwhile, a monolayer of epitaxial graphene has been grown and gently hydrogenated to reduce the coupling with the substrate^32^ (see Supplementary Information S2 for detail about the preparation of the graphene layers). Epitaxial graphene is chosen because it offers large scale layers on an insulating and transparent substrate. Backside illumination of the device is thus possible, and contrary to CVD grown graphene, no transfer step to a metal dielectric substrate is necessary. As seen from the Raman spectrum and the STM map (see Fig. 1c,d), a high quality mono-layer of graphene is obtained (see Supplementary Information S2 for details about characterization). The NPL-graphene heterostructure is built by simply drop-casting a droplet of the NPL suspension onto the patterned graphene channels using standard lithography procedure. After drying, the NPL form a uniform thick film on the graphene channels (see Supplementary Information S3 for details about the device fabrication).

As a probe for the charge transfer, we choose to use an electrolytic transistor configuration^33^,^34^. Compared to usual solid state gating method this strategy allows larger carrier density^35^ up to 10^14^ cm^−2^ which will be of utmost interest to evidence a strong charge transfer. We use a fast sweep rate (50–100 mV.s^−1^) only allowing the gating of graphene why leaving the charging state of the NPL unaffected^36^.

### Evidence for photogating

**Figure 2:**
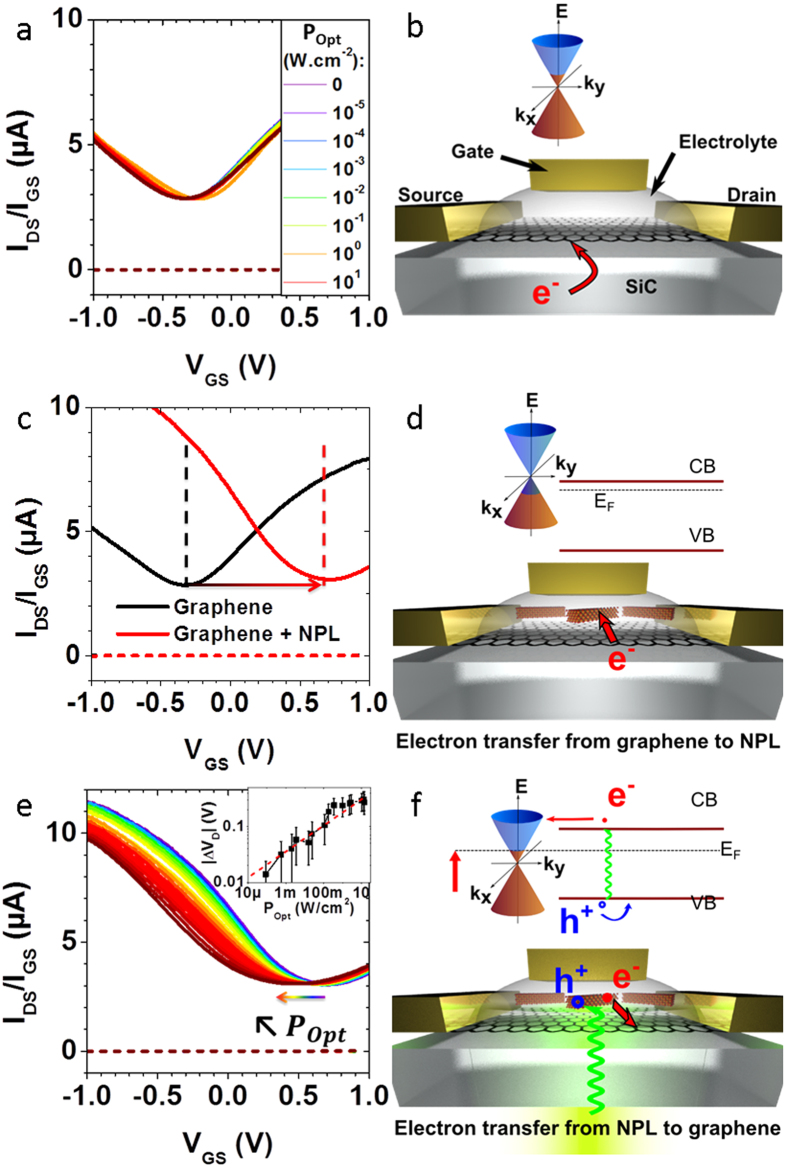
(**a,b**) Epitaxial graphene grown on insulating SiC substrate in electrolytic field effect transistor geometry. (**a**) Drain current (plain) and gate current (dashed) versus gate voltage at different laser optical powers (see inset). (**b**) Scheme of the device with gold contacts and side gate. Inset: scheme of the Dirac cone with n doped graphene. (**c,d**) Graphene decorated with CdSe nanoplatelets in the dark. (**c**) Transfer curve of undecorated graphene (black) and NPL-decorated graphene (red). The arrow shows the displacement of the Dirac point. (**d**) Scheme of the device and of the electron transfer from the graphene channel to the NPL. (**e,f**) Graphene-NPL phototransistor under illumination. (**e**) Transfer curves of the device under increasing optical intensities (see Fig. 2a). Inset: Absolute value of the Dirac point displacement *versus* optical intensity averaged over 6 samples (black squares) and a power law fit giving an exponent 0.23 (dashed red line). (**f**) Scheme of the device and of the electron transfer from NPL to graphene upon illumination.

The Dirac point of the as-prepared pristine graphene channels lies below −0.3 V (Fig. 2a), meaning that the SiC substrate induces a *n* doping of the channel (Fig. 2b). Upon back-side illumination with a 532 nm Laser with optical power density ranging from 10 μW/cm^2^ to 10 W/cm^2^, no current modulation is observed regardless of the gate voltage, see Fig. 2a. This is consistent with previous works reporting the electrical insensitivity of graphene toward light because of its low 2% absorption^37^ and the sub-picosecond recombination of the electron-hole pair^38^,^39^. CdSe NPLs are drop-casted on a bare graphene channel, and the electrolyte is then subsequently brushed (see Fig. 2d for a scheme of the device). Compared to pristine graphene, the Dirac point of the NPL-graphene hybrid shifts toward positive voltage (Fig. 2c), meaning that electrons from graphene are transferred to the NPL owing to the *n* type of these nanocrystals, the presence of electron surface traps^33^ and the quasi-resonance of NPL conduction band *versus* graphene Fermi level (Fig. 2d and Supplementary Information Tables S1 and S3). The positive shift of the Dirac point reflects a carrier density injection in the NPL film around 10^13^ charges.cm^−2^ (see Supplementary Information S4 for details about the calculation). Upon illumination, the Dirac point shifts back toward negative voltages (Fig. 2e). This evidences a charge transfer from the nanoplatelets to graphene upon illumination (Fig. 2f). Because the conduction band of the NPL (−4.4 eV, see Supplementary Info Table S3) is almost resonant with the Fermi level of graphene (−4.5 eV, see Supplementary Info Table S1), the photogenerated electrons are transferred to the graphene channel while the holes remain trapped inside the nanocrystals. This selective charge transfer thus prevents the recombination of the electron-hole pairs inside the NPL or in the graphene channel, which increases dramatically the gain of the photodetector^18^. According to reference 40, we could relate the Dirac point voltage*V*_*D*_ to the graphene carriers concentration *n* and the Fermi level position *E*_*F*_ relative to undoped graphene by


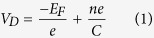


where C is the surface capacitance of the electrolyte estimated to be *C* = 2 ± 1 × μ*F*.*cm*^−2 ^34,40. Knowing the dependence of the Fermi level change upon charge carrier density (*n*)


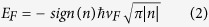


where 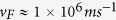 is the Fermi velocity in graphene and 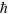 is the reduced Planck constant, we can compute the Fermi level position and the absolute carrier density in graphene (see Supplementary Information S4 and Table S1). Thus, upon 10 W/cm^2^ illumination, the negative Dirac point shift corresponds to a Fermi level change from −4.5 eV to −4.3 eV, that is to say that about 10^12^ electrons.cm^−2^ are transferred from the NPL to the graphene channel. As a comparison, under the same illumination condition, 10^9^ charges.cm^−2^ are generated in a film of NPL (see Supplementary Information Table S1). This 3 orders of magnitude difference for photogenerated charges density in comparison with the graphene hybrid evidences a photogating mechanism^13^. From the inset of Fig. 2e, it is observed that the displacement of the Dirac point *versus* the optical intensity *P*_*Opt*_ follows a power law^14^,^41^,^42^,^43^ 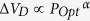 with *α* = 0.23, and tends to saturate at powers higher than 1 W/cm^2^.

### Charge transfer mechanism

In a steady state regime, the number Δ*n* of photocarriers injected from the NPL to graphene can be written as^44^:


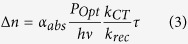


where *α*_*abs*_ is the nanoplatelet integrated absorption coefficient, *P*_*Opt*_ the optical power density, *h* the Planck constant, *v* the photon frequency, *k*_*CT*_ the charge transfer rate from nanoplatelet to graphene, *k*_*rec*_ the recombination rate inside the nanoplatelet (radiative and non-radiative^45^), and τ the lifetime of the injected carriers in the graphene (which is the same as the lifetime of trapped photocarriers in the nanoplatelets). The strength of the photogating effect thus depends on the three parameters *k*_*CT*_, τ and *k*_*rec*_. The lifetime τ is related to the nanocrystal surface chemistry and its environment, while the charge transfer rate *k*_*CT*_ can be efficiently magnified by controlling the exciton binding energy. We would like to emphasise that *k*_*rec*_ and τ themselves are depending on the carrier density Δ*n*. The overall transferred carrier density is thus self-limited upon high irradiance or particularly efficient charge transfer. In other words, if *k*_*CT*_ or *P*_*Opt*_ increases, electrons are more promptly transferred to the graphene and the supplementary remaining holes in the NPL behave as additional recombination centers, leading also to a rise of *k*_*rec*_^46^,^47^.

**Figure 3:**
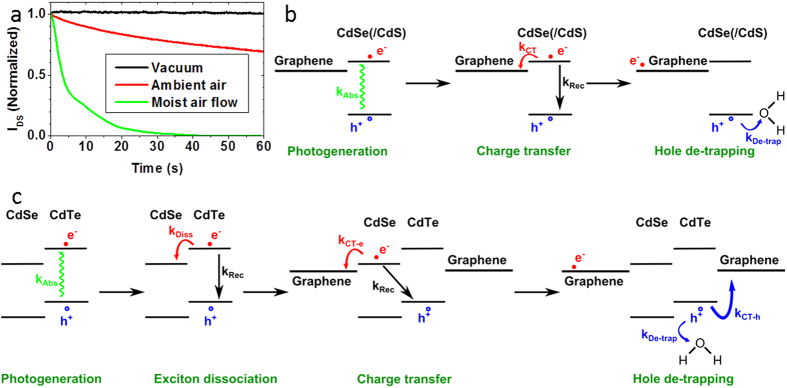
(**a**) Normalized photoresponse decay of a CdSe core NPL decorated graphene channel under various gas environments. Except for the measure under a moist air flow, the curves represent the current decay just after shutting the laser off under secondary vacuum (dark) and ambient air (red). As no photoresponse is measured under moist air flow, in this case the curve represents the photocurrent decay just after turning on the moist air flow (green). For all measurements, the curves are normalized by the photocurrent. (**b**) Proposed mechanism for electron transfer to graphene and water-assisted hole trapping for CdSe core and CdSe/CdS core/shell NPL decorated graphene upon illumination. (**c**) Proposed mechanism and associated transfer rates for the exciton dissociation, charge transfer to graphene and water-assisted hole trapping for CdSe-CdTe core-crown NPL decorated graphene. (**b,c**) *k*_*Abs*_: exciton generation rate; *k*_*Rec*_: exciton recombination rate; *k*_*CT*−*e*/*h*_: electron/hole charge transfer rate to graphene; *k*_*De*−*trap*_: water-assisted hole de-trapping rate; *k*_*Diss*_: exciton dissociation rate (core-crown NPL only).

To elucidate the mechanism determining τ, time-resolved photoresponse recovery measurements for a CdSe NPL decorated graphene were performed under various environment conditions (Fig. 3a). Under vacuum, the photoresponse is persistent upon extinction of the laser, with *τ* ≫100s (Fig. 3a-black curve). The dark signal is only recovered under ambient air (Fig. 3a-red curve). Without shutting the laser off, the dark current can even be recovered with the introduction of moisture in the system (Fig. 3a-green curve). This suggests that water is directly responsible for the recombination of trapped holes in the nanocrystals. Upon illumination and in the absence of moisture, the photogenerated holes are permanently trapped, thus increasing their lifetime and the photogating gain^20^,^48^. However, in presence of water provided by air, the surface holes get de-trapped by water, thus decreasing τ (see last step of Fig. 3b,c). In the extreme case of very moist air (Fig. 3a-green curve), no photoresponse is measured. This surface-assisted mechanism indicates that the hole traps are likely located in the sulfide-rich surface of the nanoplatelets. We would like to emphasise that this behaviour is fully consistent with previously reported transport and spectroscopic studies on the air/moisture effect on fluorescence^46^,^49^,^50^,^51^.

Apart from increasing τ, stronger photogating effect is possible by increasing the charge transfer rate *k*_*CT*_ process between illuminated NPL and graphene. This requests a deeper understanding of the coupling between the semiconductor NPL and the graphene. According to Marcus Theory^52^,^53^, the charge transfer rate of an electron (resp. hole) to the graphene channel can be estimated by


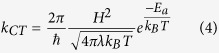


where *H* is the matrix element of the Hamiltonian coupling between an electron (resp. hole) in the conduction band (resp. valence band) of the NPL and the graphene, *λ* the reorganization energy and *E*_*a*_ the activation energy. The latter can be written


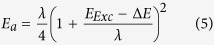


*E*_*Exc*_ being the exciton binding energy and Δ*E* = *sign*(*charge*)×(*E*_*F,Graphene*_−*E*_*CB*(*VB*),__*NPL*_) the difference between graphene Fermi level and the conduction (resp. valence) band of NPL. Faster charge transfer consequently requests to combine a weaker exciton binding energy with almost resonant coupling of one type of carrier to graphene. The second band of the semiconductor has to stay out of resonance to avoid charge recombination within the graphene. As proposed for quantum well structures, we demonstrate that the use of a heterostructure is well suited to achieve charge displacement within the semiconductor^54^. We eventually used these heterostructures as a way to enhance the electronic coupling between graphene and the nanocrystals.

**Figure 4:**
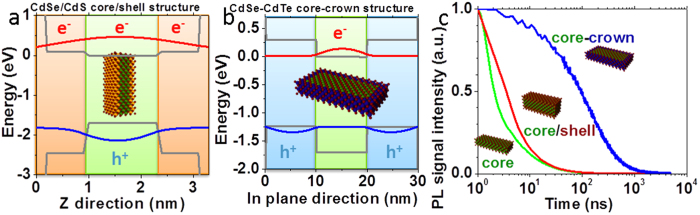
(**a**) Calculated band structure of the CdSe/CdS core/shell heterostructure. Grey: energy levels of the bulk materials, red (resp. blue): conduction (resp. valence) band of the heterostructures. Holes are confined in the core while electrons are delocalized over the entire structure. (**b**) Calculated band structure of the CdSe-CdTe core-crown type II heterostructure. Grey: energy levels of the bulk materials, red (resp. blue): conduction (resp. valence) band of the heterostructures. Holes are confined in the crown while electrons are confined in the core. (**c**) Photoluminescence decay spectra of CdSe core NPL (green), CdSe/CdS core/shell NPL (red), and CdSe-CdTe core-crown NPL (blue).

CdSe NPLs with their 2D aspect and limited static dielectric (ε = 10) constant^55^ have a large exciton binding energy (250 meV typically^55^,^56^) which prevents an efficient charge dissociation^7^,^8^. A key advantage of colloidal grown 2D nanoplatelets compared to other transition metal dichalcogenides system is the possibility to further grow at the atomic scale some heterostructures perpendicular to the NPL plane (core/shell heterostructure) or into the plane (core-crown heterostructure). On 4 monolayers (ML) CdSe core NPL in non-polar solvent, we use the c-ALD procedure^33^ to directly grow in the colloidal suspension a CdS shell (0.9 nm of CdS on each side) around the CdSe cores. In this core/shell heterostructure, there is a complete delocalization of the electron wavefunction over the whole thickness, while the hole stays confined into the CdSe core, see Fig. 4a. We also grew CdSe-CdTe core-crown NPL. The CdSe cores in suspension are expanded laterally by growing a 10 nm large CdTe crown, while keeping the thickness unchanged^28^. In this material, the electron wavefunction is localized within the CdSe core, while the holes get localized into the crown (type II band alignment), see Fig. 4b. This heterostructure thus enables the exciton dissociation at the nanocrystal scale before charge transfer to graphene^57^, see Fig. 3c. Apart from the improved charge dissociation, the introduction of a CdTe crown is also motivated by its bands shifted closer to the vacuum level. This shift raises the valence band level closer to the graphene Fermi level while the conduction band is getting off resonance, see Fig. 3c. As a consequence, for the CdSe-CdTe core-crown heterostructure, we expect a more favourable hole transfer to graphene. The two types of heterostructures present a reduced overlap of the electron and hole wavefunctions and thus likely a reduced exciton binding energy. Details about the synthesis are given in Supplementary Information S1. The spectra of suspensions of such heterostructures are presented in Fig. 1a and confirm the successful growth of core/shell and core-crown NPL^27^,^28^.

To quantify the reduction of the exciton binding energy, we use time resolved photoluminescence (PL) spectroscopy, see Fig. 4c. We can indeed relate the radiative lifetime to the exciton binding energy *E*_*Exc*_ and the oscillator strength*f*thanks to the relation 6^58^,^59^


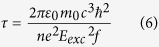


with *ε*_0_ the vacuum permittivity, *m*_0_ the rest mass of the electron, 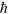 the reduced Planck’s constant, *n* the optical index of the medium. The oscillator strength can be estimated as


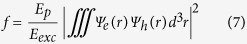


*E*_*p*_ being the Kane parameter and *Ψ*_*e/h*_ the electron or hole wavefunction. Defining


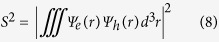


as the overlap integral, the radiative lifetime is now given by


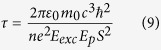


In order to estimate the S value, we use a two bands k.p. model^60^,^61^. More details are given in the Supplementary Information S6 and Table S4 give the parameters used for this modelling. Compared to the core NPL, the core/shell heterostructure has an overlap integral reduced by ≈10%, while for the core-crown heterostructure the overlap decreases by about two orders of magnitude. Since the exciton lifetime is 2.5 times longer for the core/shell NPL (see Fig. 4c), we can roughly estimate that its *E*_*Exc*_ value is twice smaller than for core only NPL (Supplementary Information S6).

**Figure 5:**
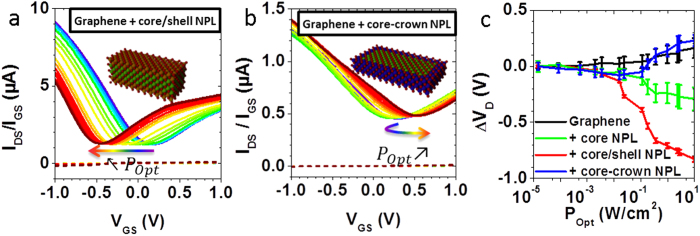
Transfer curves under increasing illumination power (see Fig. 2a for the legend) for (**a**) CdSe/CdS core/shell NPL – graphene phototransistor; (**b**) CdSe-CdTe core-crown NPL – graphene phototransistor. (**c**) Displacement of the Dirac point voltage *versus* incident optical power for graphene (black), graphene – CdSe core (green), graphene – CdSe/CdS core/shell (red), graphene-CdSe-CdTe core-crown (blue). The points are averaged over 3 to 6 devices, and the error bars represent the standard deviation to the mean.

To confirm that the charge transfer to graphene can be tuned by engineering a heterostructure at the nanoplatelet scale, hybrid graphene-NPL phototransistors involving these heterostructured nanocrystals have been fabricated. An identical procedure than for core-only NPL-graphene hybrids was used to pattern the graphene channels, deposit the NPL film and fabricating the electrolyte gated transistors. As expected from the reduction of the exciton binding energy, we notice that under illumination, the Dirac point shifts to more negative voltage for the hybrid involving core/shell NPL than for core-only NPL (Fig. 5a,c). The amount of transferred electrons is then about 3 times larger (see Supplementary Information Table S1).

Knowing the exciton binding energy *E*_*Exc*_, the self-exchange reorganization energy λ and the energy difference between the NPL conduction band the graphene Fermi level Δ*E*, we can quantify the improvement of the charge transfer rate *k*_*CT*_ for core/shell NPL in comparison to core only NPL. The self-exchange reorganization energy was recently evaluated^62^ for a film of colloidal quantum dot and was shown to poorly depend on the nanoparticle geometry. The NPL contribution to the λ is thus estimated to be 0.1 ± 0.05 eV. Since the reorganization energy for graphene nanoribbons and for graphene-like π-conjugated systems sharply decreases as a function of the number of carbon atoms^63^,^64^,^65^, the graphene contribution to λ is largely smaller than 0.1 eV and can be neglected. The energy level difference Δ*E* remains equal to 0.1 eV since the 0.2 eV Fermi level shift between core-only and core/shell NPL decorated graphene reflects the confinement energy reduction of 0.2 eV between these two types of NPL (see Supplementary Information S4 and S5). The reduction of *E*_*Exc*_ from 0.2 eV to 0.1 eV while switching from core to core/shell NPL thus cancels the energy barrier *E*_*Exc*_−Δ*E*. The activation energy *E*_*a*_ is thus reduced by a factor 4, leading to a potential increase of the charge transfer rate *k*_*CT*_ up to a factor 150 at room temperature. Because a more efficient charge transfer from NPL to graphene also means a more charged nanocrystal and thus more Auger recombinations, the recombination rate *k*_*rec*_ is also increased and the trapped carrier lifetime *τ* is lowered. When considering the expression of injected carriers in graphene


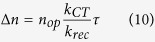


with *n*_*op*_ the sheet density optically generated in the covering semiconductor, we expect that the increase of the charge transfer rate is partially compensated by the opposite evolution of *τ*/*k*_*rec*_ at steady state. However, the net increase of the injected carriers Δ*n* in graphene by switching from core-only to core/shell NPL validates the exciton binding energy engineering strategy.

In the case of core-crown NPL decorated graphene under illumination higher than 100 mW.cm^−2^, the Dirac point shift reverses toward positive voltages, meaning that holes are being injected to the graphene (Fig. 5b,c). This is consistent with the rise in absolute energy of the valence band of CdTe compared to CdSe (see Supplementary Information Table S3). When the electron injection from the CdSe core saturates, holes from the CdTe crown are efficiently injected into the graphene channel.

For all materials, the shift of the Dirac point with illumination is sublinear and follows approximately a power law with a 0.23 exponent. This behaviour is also a consequence of the dependence of the recombination rate *k*_*rec*_ and the trapped carrier lifetime *τ* on the number of injected carriers in grapheme Δ*n*. A higher photon flux means a higher optically generated electron-hole pairs density *n*_*op*_. As for *k*_*CT*_, the resulting increase of injected carriers Δ*n* is moderated by the opposite evolution of *τ*/*k*_*rec*_. This leads to the observed sublinear dependence of the charge transfer on incident light power at steady state.

### Device performances

**Figure 6:**
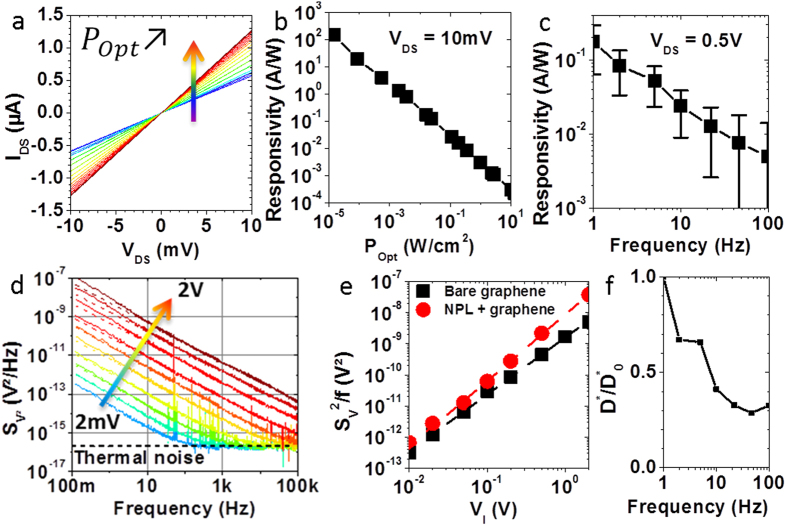
(**a–c**) CdSe/CdS core/shell NPL decorated graphene samples. (**a**) Current *versus* voltage of a single channel under increasing illumination (see Fig. 2a-left for the legend). (**b**) Responsivity *versus* optical power density for *V*_*DS*_ = 10*mV*. (**c**) Responsivity as a function of optical modulation frequency. (**d**) Square voltage noise spectral densities of a bare graphene channel under different Wheatstone bridge excitation voltages *V*_*I*_. Dashed line: fit according to equation 
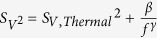
. (**e**) Noise level *βversus* excitation voltage*V*_*I*_ of bare graphene (black) and CdSe/CdS NPL decorated graphene (red). Dashed line: power law fit giving an exponent1.9 ± 0.1. (**f**) Reduced specific detectivity 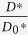 where 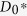 is the specific detectivity at 1Hz for a CdSe/CdS core/shell NPL decorated graphene photodetector as a function of frequency. Details about the calculations are given in Supplementary Information S7.

Being the best candidate for photodetection among the tested materials, we made a photodetector by depositing CdSe/CdS core/shell nanoplatelets on a graphene channel. Current *versus* voltage characteristics are measured under increasing illumination power *P*_*Opt*_, see Fig. 6a. An increase of the current is measured under increasing illumination, arising from the conductance modulation of the graphene channel due to photogating (Fig. 5a). We verified that no significant modulation is measurable for bare graphene devices (Supplementary Information Figure S4a). The responsivity figure of merit


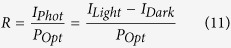


is mainly driven by the illumination power (Fig. 6b). This is due to the weak dependence of the photocurrent to the optical intensity, allowing the measurement of weak optical signals^13^. The responsivity under modulated illumination is also measured (Fig. 6c and Supplementary Information S7 for details about the measurement procedure). For modulation frequencies ranging from 1 Hz to 100 Hz, the responsivity decreases with the modulation frequency. These behaviours are consistent with the fact that the system is a photogating-based graphene photodetector. This strategy indeed allows enhanced photogain


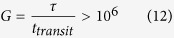


because of long photocarrier lifetime *τ*>1*s* due to efficient hole trapping and also thanks to the short transit time *t*_*transit*_ ≈1 μs made possible by the high mobility *μ* ≈ 1000 cm^2^.V^−1^.s^−1^ of graphene^32^. It comes at the price of a lowered bandwidth, the latter being inversely proportional to the carrier lifetime τ.

The photodetection performances of photodetectors can be compared by calculating the specific detectivity D* figure of merit. Since it can be expressed as


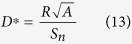


where *A* the optical area and *S*_*n*_ the current noise spectral density, a measurement of the noise is required. We emphasise that the latter could not be estimated by the Johnson-Nyquist (thermal) noise nor by the shot noise because graphene possesses 1/f excess noise at frequencies below a few kHz^66^. It is still an open question whether the presence of nanocrystals decorating the graphene may lead or not to a dramatic enhancement of the noise. In order to measure the noise arising from a graphene channel, we chose a Wheatstone bridge configuration comprising of 4 identical graphene channels biased using a battery (see Supplementary Information S3 scheme S1)^67^. The fluctuations of the voltage difference *V* between the middle of the two branches are the averaged noise coming from the 4 graphene channels, *i.e*. 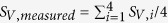. This strategy suppresses the upstream noise arising from the source and common-mode noise arising from environmental perturbations. The square-voltage noise spectral density is measured for a bare graphene channel under increasing bridge excitation voltage *V*_*I*_ and presented in Fig. 6d. A 3 parameters fit according to the equation 14


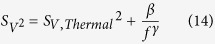


is performed on each measurement. The obtained thermal noise 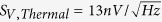 is in good agreement with the average resistance of the channels *R* = 8*k*Ω. The value of the power exponent is *γ* = 1.05 ± 0.05 as expected for 1/f noise without generation-recombination bulges. The magnitude β of the noise is plotted *versus* the applied voltage bias in the Fig. 6e-black curve. A simple 3 parameters power law fit 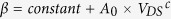 is performed, where *V*_DS_ = *V*_*I*_/2 is the voltage applied on each graphene channel. This gives an exponent value *c* = 1.9 ± 0.1 and a coefficient *A*_0_ = 6.5 ± .5×10^−9^, consistent with previous report of 1/f noise in graphene^66^. The square voltage noise spectral density 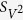 thus scales quadratically with the applied bias. In the framework of the Hooge model, knowing the total number of carriers involved in transport *N*, we can calculate the α parameter characterizing the intrinsic noise of a sample:


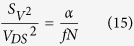


The channel area being 270 × 30 μm^2^ and the carrier density of bare graphene on SiC being 8.10^12^ cm^−2^ (Supplementary Information Table S1), we estimate the value of the Hooge parameter *α* = *A*×*N* = 4 ± 1.

CdSe/CdS core/shell nanoplatelets are then deposited on the four channels of the Wheatstone bridge, and the same noise measurements are performed (see Supplementary Information Figure S4b and Fig. 6e - red curve). The 1/f behaviour is still maintained and the thermal noise floor is reached. However, the noise level is increased by a factor 3, meaning that *α*/*N* = 20 ± 2 × 10^−9^.

According to a carrier density of graphene after CdSe/CdS NPL deposition of about 3.10^12^ cm^−2^ (Supplementary Information Table S1), this gives a Hooge parameter *α* ≈ 5. Given the accuracy of the *α* parameter estimation, this parameter is barely affected by the deposition of nanoplatelets on graphene. This is compatible with a noise modulation resulting from the graphene carrier density modulation upon NPL deposition^68^. Rather than defect centers, nanoplatelets could possibly behave as a passivation layer toward air^69^. To the best of our knowledge, this is the first time that the effect of particles deposition on graphene is reported in term of noise. Measuring noise in nanocrystal-based devices is of crucial importance since 1/f noise cannot be predicted despite being ubiquitous, thus limiting devices ultimate performances^66^,^67^.

The detector achieves a specific detectivity D^*^ = 10^6^ Jones weakly dependent on the modulation frequency (Supplementary Information S7 and Fig. 6f), owing to the similar dependence of the noise spectral density and responsivity *versus* frequency of operation. Compared to PbS-graphene devices, our all 2D hybrid achieves relatively low detectivities^13^. This is a direct consequence of the reduced exciton binding energy for PbS quantum dots, around 10 meV thanks to their large dielectric constant^70^. Since TMDC together with chalcogenides nanoplatelets exhibit high exciton binding energies over hundreds of meV resulting from the material and their monolayer structure^21^,^22^,^23^, this emphasises the need to reduce the exciton binding energy to build efficient monolayer based devices. Our results suggest that it could be practically done by building heterostructures at the monolayer scale.

## Conclusions

Heterostructures grown at the nanocrystal scale is a practical strategy to 1) lower the exciton binding energy by delocalizing one type of carrier *versus* the other one, 2) choose the availability of one or the other type of carrier toward charge transfer. Applied to a photodetector made from CdSe-based nanoplatelets decorated on a graphene channel, CdS-shell covered NPL induce a more efficient *n*-type photogating of the graphene while CdTe-crown surrounded NPL produce a *p*-type photogating. We expect this strategy to be profitable to other 2D materials^71^, especially heterostructures involving TMDC because of their giant exciton binding energies. Noise measurements reveals that the deposition of nanoplatelets on graphene increases the noise magnitude by a factor 2–3 but does not alter the 1/f behaviour nor the Hooge’s parameter α.

## Additional Information

**How to cite this article**: Robin, A. *et al*. Engineering the Charge Transfer in all 2D Graphene-Nanoplatelets Heterostructure Photodetectors. *Sci. Rep*. **6**, 24909; doi: 10.1038/srep24909 (2016).

## Supplementary Material

Supplementary Information
