# Supplementary material for: Engineering the Charge Transfer in all 2D Graphene-Nanoplatelets Heterostructure Photodetectors
Source: Sci Rep. 2016 May 4;6:24909. doi: 10.1038/srep24909 (PMC4855231; doi:10.1038/srep24909)
Supplement: Supplementary Information [file srep24909-s1.pdf]

Supplementary Information for Engineering the Charge Transfer in all 2D Graphene-Nanoplatelets Heterostructure Photodetectors

A. Robin [1,2], E. Lhuillier [2,3], X. Z. Xu [1], S. Ithurria [1], H. Aubin [1], A. Ouerghi [4], and B. Dubertret [1,]**

[1]Laboratoire de Physique et d'Étude des Matériaux, PSL Research University, CNRS UMR 8213, Sorbonne Universités UPMC Univ Paris 06, ESPCI ParisTech, 10 rue Vauquelin, 75005 Paris, France

[2]Nexdot, 10 rue Vauquelin, 75005 Paris, France

[3]UPMC-CNRS, UMR 7588, Institut des Nano-Sciences de Paris (INSP), 4 place Jussieu, 75005 Paris, France

[4]Laboratoire de Photonique et Nanostructures, CNRS, Route de Nozay, 91460, Marcoussis, France

*E-mail: emmanuel.lhuillier@insp.upmc.fr and benoit.dubertret@espci.fr.

Contents

1. Chemicals and precursors 2
Metal/ions precursors 2
Solvents 2
Ligands 2
Electrolyte 3
Preparation of $Cd(Myr)_2$ 3
CdSe core nanoplatelets 3
CdSe/CdS core/shell nanoplatelets 3
CdSe-CdTe core-crown nanoplatelets 4
Material characterization 5
2. Graphene growth and channels patterning 6

Epitaxial graphene on 4H-SiC 6
Patterning in graphene channels 6
3. Devices........ 7
Device fabrication........ 7
Electrical characterization 8
Noise measurement setup 8
Fequency photoresponse setup 9
4. Charge density and Fermi level shift computation 10
For a NPL film 10
For a graphene-NPL heterostructure 10
5. Determination of the nanoplatelet band parameters 12
6. Determination of exciton binding energy 13
Wavefunction calculation........ 13
Exciton binding energy calculation........ 13
7. Performances of CdSe/CdS NPL decorated graphene photodetector 15
8. Effect of the nanoplatelets film thickness on the photoconduction properties........ 17
9. References........ 18

1. Chemicals and precursors

All chemical are used as received.

Metal/ions precursors

Se powder (Sigma-Aldrich, 99.99%), 60 mesh Te powder (99.999%, Strem Chemicals), Cadmium oxide (CdO, Sigma-Aldrich 99.99%), Cadmium nitrate ($Cd(NO_3)_2(H_2O)_4$, Sigma-Aldrich 99.999%), Cadmium acetate ($Cd(OAc)_2(H_2O)_2$, Sigma-Aldrich 98%), Sodium hydroxide (Sigma-Aldrich, 97%).

Solvents

n-Hexane (VWR, 98%), ethanol (Carlo Erba, 99.5%), n-Methyl formamide (NMFA, Sigma-Aldrich, 99%), Octadecene (ODE, Sigma-Aldrich 90%), TOP (90%, Sigma-Aldrich).

Ligands

Sodium sulfide nonahydrate ($Na_2S,9H_2O$, Sigma-Aldrich 98%), Myristic acid (Sigma-Aldrich, 95%), Oleic acid (OA, 99%).

Electrolyte

$LiClO_4$ (Sigma-Aldrich, 98%), Polyethylene glycol M_W=6x10^3 g.mol^{-1} (PEG 6k) (Fluka).

In a glove box, 50 mg of $LiClO_4$ are mixed in 230 mg of polyethylene glycol (M_W = 6 kg.mol^{-1}) at 150°C on a hot plate in a glove box. Once the mixture looks homogeneous, the solution is cooled down and kept in the glove box.

Preparation of Cd(Myr)$_2$

In a 1 L Erlenmeyer flask, 3.2 g (80 mmol) of NaOH are dissolved in 500 mL of methanol. To this solution are added 18.2 g (80 mmol) of Myristic acid. The solution becomes clearer but white aggregates remain. It is stirred for 15-20 minutes at room temperature. Meanwhile, 8.2 g (26 mmol) of Cadmium nitrate tetrahydrate are dissolved in 50mL of methanol. This solution is added to the sodium myristate solution. A white precipitate is formed very rapidly. After 15 minutes of stirring at room temperature, the white solid is isolated by filtration, washed three times with methanol and dried under vacuum overnight.

CdSe core nanoplatelets

CdSe nanoplatelets core are synthetized using the procedure described in reference 1. In a 100 mL three-neck flask, 240 mg of Cd(Myr)$_2$ and 25 mg Se powder are mixed in 30 ml ODE. The solution is degassed under vacuum for 20 min at room temperature. Under Argon, the temperature is set to 240°C. At 204°C, 160 mg of Cd(OAc)$_2$ is quickly added. The reaction is performed 12 min at 240°C. 1 ml of oleic acid is quickly injected to quench the reaction and the solution is cooled down to room temperature. The precipitation of the NPL is done by adding ethanol. After centrifugation the obtained solid is redispersed in hexane. The cleaning procedure is repeated three times. The obtained objects have a typical lateral extension of 10 nm x 40 nm (see Figure 1b). The NPL have a narrow first excitonic feature centred at 510 nm (see Figure 1a).

The resulting nanoplatelets are capped with long and insulating oleic acid ligands. These are exchanged with sulfide S^{2-} ligand using the procedure given in reference 2. Briefly, it is the first step of the growth of a CdS shell described in the next paragraph, and relies on a transfer to a polar phase containing the sulfide ligands. The final objects have similar dimensions than the cores, but have a first excitonic feature around 540 nm (see Figure 1a).

CdSe/CdS core/shell nanoplatelets

The CdS shell is grown using the low temperature colloidal atomic layer deposition growth described in reference 3. CdSe core nanoplatelets in hexane are transferred in NMFA by addition of sodium sulfide nonahydrate in NMFA. It is equivalent to the growth of a half layer. The hexane supernatant is removed, and the resulting CdSe/S^{2-} nanoplatelets are then precipitated with acetonitrile to remove the excess of

sulfide and dispersed in NMFA. The second half layer is grown by addition of $Cd(OAc)_2$ in NMFA (the reaction is quasi instantaneous), the excess of precursors is removed by precipitating the nanocrystals with a mixture of toluene and acetonitrile (5:1). The $CdSe/S/Cd^{2+}$ nanoplatelets are eventually dispersed in NMFA. It is possible to grow several layers by using Na_2S and $Cd(OAc)_2$ as precursors of sulfide and cadmium. Here the NPL present 3.5 layers of CdS: $CdSe/(CdS)_{3.5}$.

The obtained objects have a typical thickness of 5 nm (3 monolayers of CdS on each side of the NPL) with a lateral extension of 10 nm x 40 nm (see **Figure S1**). The first excitonic feature is around 630 nm (see Figure 1a).

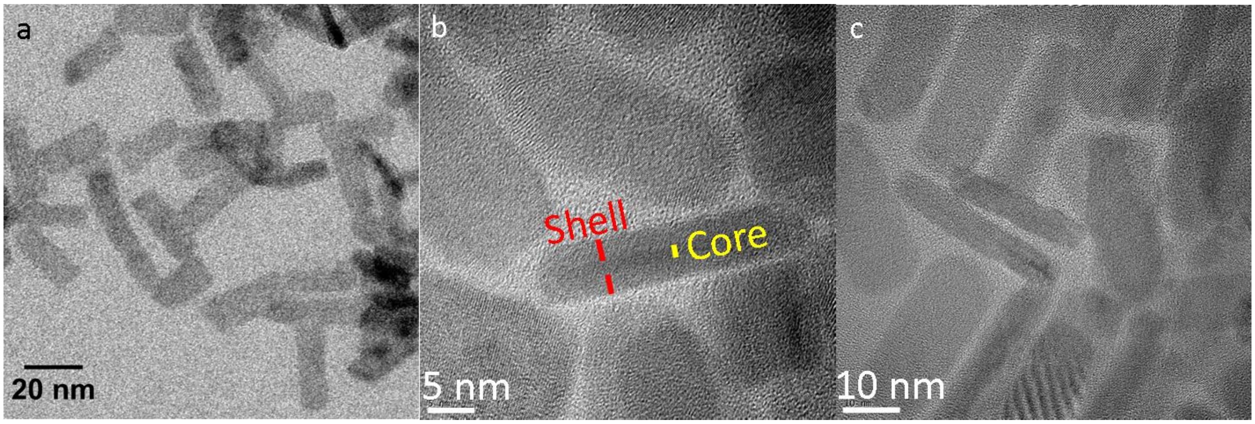

Figure S1. (a) TEM image of core/shell CdSe/CdS nanoplatelets. (b,c) HRTEM images of the same NPL evidencing the core/shell heterostructure.

CdSe-CdTe core-crown nanoplatelets

The core-crown nanoplatelets synthesis is slightly modified from reference 4. 2ml of CdSe core nanoplatelets in hexane are precipitated with ethanol, resuspended in 5 mL of ODE, and transferred into a 25 mL three-neck flask. 170 mg of $Cd(Myr)_2$ (0.3 mmol) and 55 mg of $Cd(OAc)_2$ (0.2 mmol) are added and the mixture is degassed under vacuum for 30 min. The temperature is then increased under an Argon flow, and when it reached 235°C, a solution of 50 µL of 1 M TOPTe in 1 mL of ODE is added at an injection rate of 60 mL/h. After 15 min, 1 mL of OA is swiftly added, and the mixture is cooled to room temperature. The core-crown NPLs are purified by selective precipitation with hexane and ethanol followed by centrifugation at 5000 rpm for 10 min.

Their lateral extension are typically 50 nm x 40 nm (see **Figure S2**a), and they mostly present the excitonic feature of the CdTe crown around 558 nm (see Figure 1a), the CdSe core first excitonic feature should be around 510nm which also correspond to the second excitonic of the CdTe crown. As for the CdSe core NPL, they are initially capped with long ligands, and are exchanged with sulfide ligands with the exactly same procedure than for the CdSe cores. Their lateral dimensions remain unchanged, but the excitonic features broaden and redshift toward 600 nm for the CdTe crown.

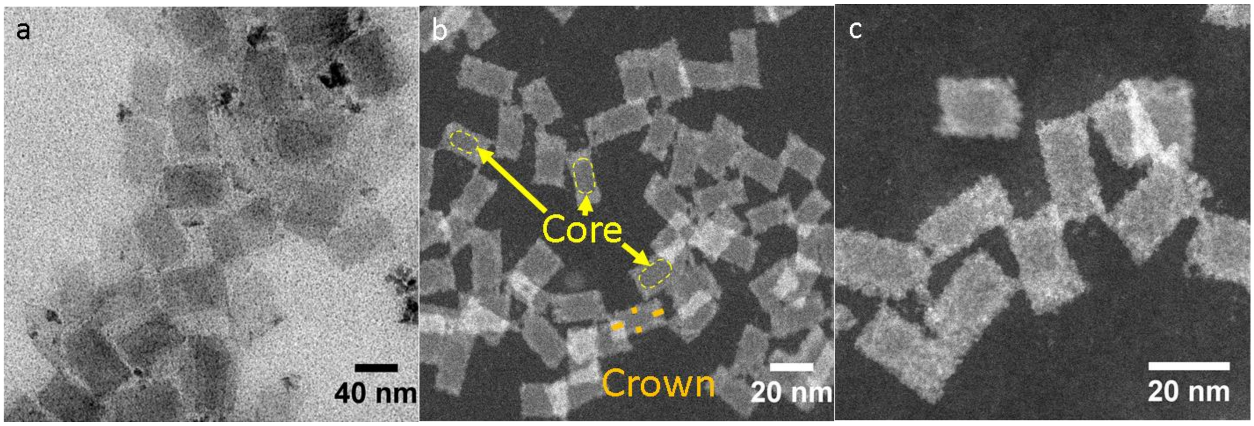

Figure S2. (a) TEM image of core-crown CdSe-CdTe nanoplatelets. (b,c): HAADF-TEM images of core-crown NPL at the beginning of the crown growth evidencing the core-crown heterostructure.

Material characterization

Transmission electron imaging is done using a JEOL 2010 microscope. Scanning electron microscopy is performed with a FEI Magellan. XRD diagrams are acquired on a Philips X'pert system. UV-visible absorption is done with a Cary 5E. Photoluminescence spectra are measured using a Jobin Yvon Fluromax 3 and photoluminescence lifetimes are measured with an Edinburgh F900.

2. Graphene growth and channels patterning

Epitaxial graphene on 4H-SiC

The single layer graphene studied in this paper is produced *via* a two-step process from the SiC face of a 4H-SiC(0001) substrate. Prior to graphitization, the substrate is hydrogen etched (100% H_2) at 1550°C to produce well-ordered atomic terraces of SiC. The terraces cover the whole substrate (about 1 cm^2), with a typical step height of about 4–6 nanometers. The SiC sample is heated to 1000°C in a semi UHV and then further heated to 1525°C in an 800 mBar Ar atmosphere for 10 minutes. This graphitization process results in the growth of an electrically active graphene layer on top of the interface layer. The hydrogenation of the graphene samples is carried in the same furnace than for the epitaxial growth out using 100% H_2 at 820°C for 10 minutes.[5] The graphene sample is characterized using Raman spectroscopy and Scanning Tunneling Microscopy. The micro-Raman spectroscopy is performed at room temperature with a Renishaw spectrometer using 532 nm excitation wavelength lasers light focused on the sample by a DMLM Leica microscope with a 503 objective and a power of 5 mW with spot size of about 1 mm. The STM measurements were carried out using an AFM-STM Omicron under a pressure below 5×10^{-11} millibar at room temperature. STM image presented here were acquired in a constant current mode for different voltages applied to the sample. According to the $55\ \mathrm{cm}^{-1}$ FWHM of the 2D peak at $2715\ \mathrm{cm}^{-1}$, mono-layer of graphene is obtained with multiple-layer islands (Figure 1c). The layer is nearly defect-free owing to the low intensity of the D peak at around $1350\ \mathrm{cm}^{-1}$. The position and magnitude of the G peak is difficult to establish since it interferes with the signal coming from the 4H-SiC substrate in the $1450 - 1950\ \mathrm{cm}^{-1}$ range. Figure 1d shows the magnification of the graphene monolayer before hydrogenation, the periodicity of this honeycomb structure is equal to 2.5 Å which is in good agreement with the (1x1) graphene lattice.

Patterning in graphene channels

The graphene channels are patterned using a standard 3-steps lithography procedure. At the first step, contact pads are patterned using nLof 2070 resist. The graphene is then etched by RIE O_2 plasma etching, and Cr/Au 30/70 nm electrodes are deposited. A similar second step defines the drain, source and side-gate electrodes, without graphene etching. The final step defines the graphene channel with AZ5214E resist and O_2 plasma etching. The graphene channels are 120 or 270 µm long, and 30 µm wide.

3. Devices

Device fabrication

The graphene channels are first heated up at 100°C in the glovebox to remove loose adsorbents. If appropriate, two-three 5 µL droplets of a suspension of the desired nanoplatelets are deposited on the channels. The solvent is allowed to evaporate during 10 minutes after each drop, see Figure S3 for SEM images of such a device evidencing the graphene channel covered by a thick NPL film. For transistor measurements an electrolyte comprising Lithium Perchlorate dissolved in Polyethylene Glycol matrix is soften at 100°C and carefully brushed on the channels.[6,7] The samples are eventually cooled down to room temperature for at least two hours in the glovebox.

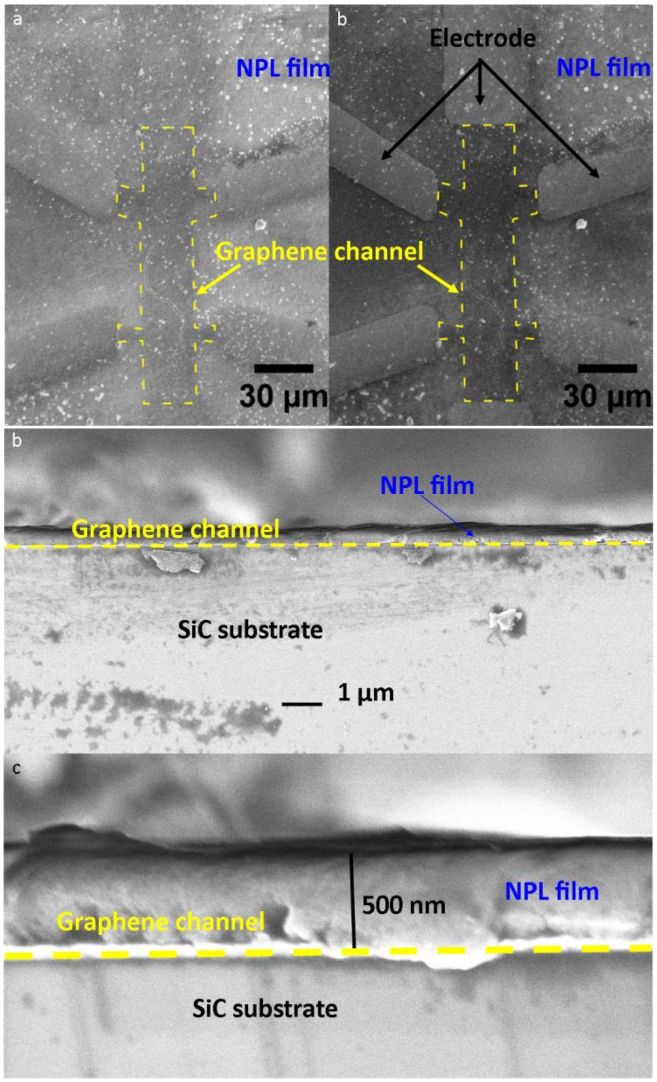

Figure S3. (a,b) SEM picture of a NPL film on a graphene channel at (a) 10 kV (both the graphene channel and the gold contacts are visible through the NPL film) and (b) 20 kV (only the contacts are visible through the NPL film). The NPL film covers the whole sample. (b,c) Cross-section SEM picture of the same NPL – graphene hybrid at two different magnifications.

Electrical characterization

Measurements are performed in air at ambient temperature.

Electrical measurements (I-V and transistor curves) are performed using a Keithley 2634b to probe and bias the drain and gate electrodes. Illumination is provided by a 532 nm DPSS laser diode controlled in current to provide optical power densities ranging from 10 µW/cm² to 10 W/cm². For the photoresponse spectrum measurement, a NKT Photonics SuperK Extreme supercontinuum lasers together with a SuperK VARIA filter are used to illuminate the sample.

Noise measurement setup

To make accurate measurements of the noise of a graphene channel, a Wheatstone bridge configuration consisting of 4 graphene channels with identical resistance is chosen, see Scheme S1. The signal difference V_O between the middle of the two branches is a measurement of the noise averaged upon 4 channels, while the up- and down-stream signals (noise and DC component) are rejected because being Common-Mode. The signal difference is amplified using a low voltage noise - high input impedance Instrument Amplifier (Analog Devices INA217) powered on batteries. The output of the amplifier is sent to a Stanford Research SR780 signal analyzer.

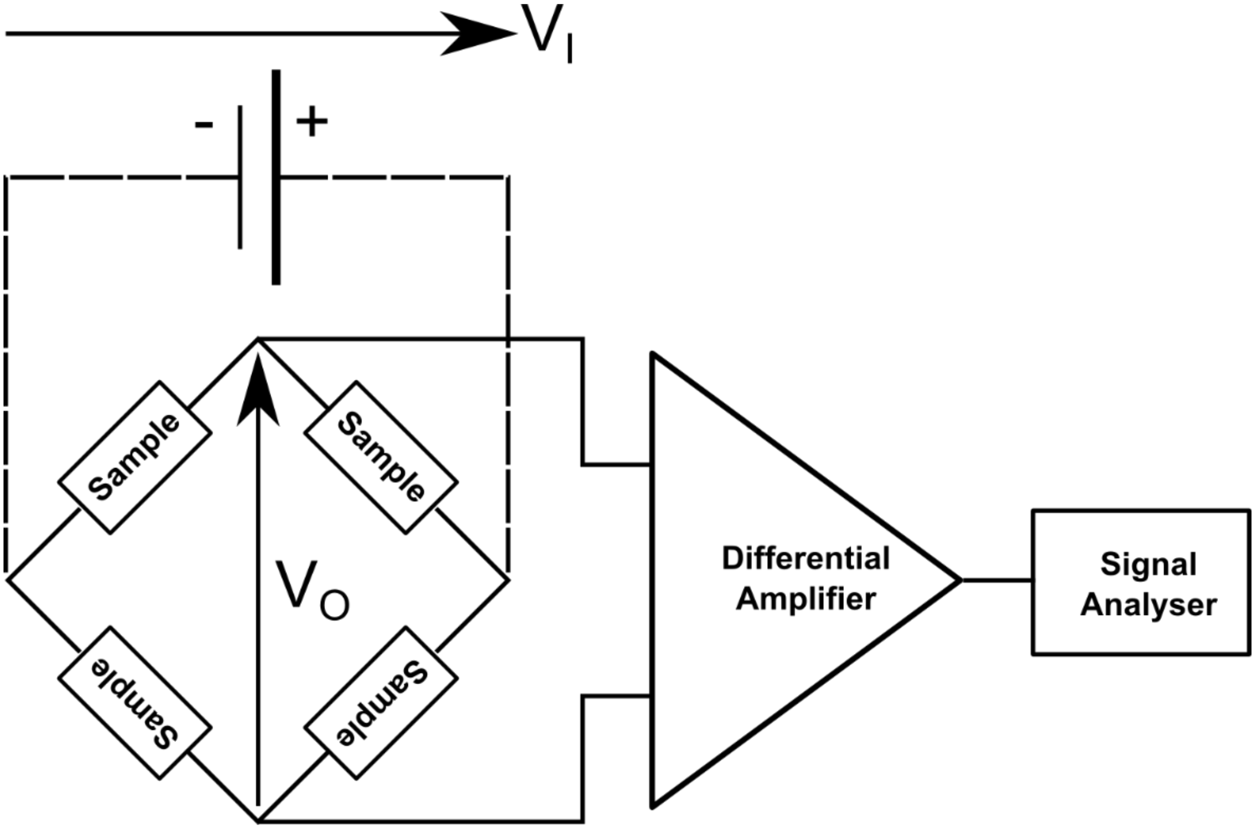

Scheme S1. Setup for noise measurement, V_O being the voltage difference between the middle of the two branches of the Wheatstone bridge and V_I being the bridge excitation voltage.

Fequency photoresponse setup

The same setup than for noise measurement is used, by directly connecting the signal analyser to the middle of the two branches of the Wheatstone bridge. A 532 nm Laser is focused on a single graphene channel, and modulated by a chopper whose frequency is adjusted between 1 Hz and 100 Hz. The peak-to-peak amplitude of the resonant mode of the response is followed *versus* chopping frequency and increasing illumination intensity.

4. *Charge density and Fermi level shift computation*

For a NPL film

Under 10 W/cm² illumination, As a comparison, under the same illumination condition, the conductivity of a film of NPL changes of $\Delta\sigma = 10^{-4}\, S/m$.[8] Knowing the mobility $\mu \sim 10^{-2}\mathrm{cm^2V^{-1}s^{-1}}$ of such film,[6,8] we could compute the photogenerated charge surface density Δn thanks to the relation

$$\Delta n = \frac{\Delta\sigma}{e\mu} \times t$$

t = 200 nm being the film thickness. This gives a generation of $10^9\mathrm{cm^{-2}}$ charges in a film of NPL.

For a graphene-NPL heterostructure

According to reference 9, we could relate the voltage of the Dirac point V_D to the Fermi level position E_F *versus* undoped graphene of graphene and its carrier concentration n (positive value means hole concentration) by

$$V_D = -\frac{E_F}{e} + \frac{ne}{C}$$

with C being the surface capacitance of the electrolyte estimated to be $C = 2.2 \times 10^{-6}\mathrm{Fcm^{-2}}$.[9] Knowing the dependence of the Fermi level change upon charge carrier density

$$E_F = -sign(n)\hbar v_F\sqrt{\pi|n|}$$

or

$$n = sign(V_D)\frac{{E_F}^2}{\pi\hbar^2{v_F}^2}$$

where $v_F = 1.1 \times 10^6\mathrm{ms^{-1}}$ is the Fermi velocity in graphene, we could compute the Fermi level change *versus* the position of pristine graphene (-4.5 eV), the absolute Fermi level position, the amount of carriers being transferred to graphene (negative values means electrons being transferred) and the absolute carrier density in graphene (negative values means electron concentration) by solving these two equations:

$$V_D = \frac{sign(n)\hbar v_F\sqrt{\pi|n|}}{e} + \frac{ne}{C}$$

$$V_D = -\frac{E_F}{e} + sign(V_D)\frac{{E_F}^2 e}{\pi\hbar^2{v_F}^2 C}$$

The results are presented in Table S1 and are averaged on 8 to 20 samples. For a given scenario, the perturbation is highlighted in bold. The Fermi level change and the transferred carrier density are thus calculated relative to the system before the perturbation.

**Scenario**	V_D **(V)**	**Fermi level change** E_F **(eV)**	**Absolute Fermi level (eV)**	**Transferred carrier density** (cm^{-2})	**Absolute carrier density** (cm^{-2})
Bare graphene deposited on SiC	-1.0	0.4	-4.1	-8×10^{12}	-8×10^{12}
CdSe core NPL deposited on graphene/SiC	0.0	-0.4	-4.5	8×10^{12}	0×10^{12}
10 W/cm² illumination on CdSe core NPL + graphene/SiC hybrid	-0.3	0.2	-4.3	-2×10^{12}	-2×10^{12}
CdSe core/shell NPL deposited on graphene/SiC	0.4	-0.2	-4.7	1×10^{13}	3×10^{12}
10 W/cm² illumination on CdSe core/shell NPL + graphene/SiC hybrid	-0.5	0.4	-4.3	-6×10^{12}	-3×10^{12}
CdSe core-crown NPL deposited on graphene/SiC	0.1	-0.5	-4.6	8×10^{12}	3×10^{11}
10 W/cm² illumination on CdSe core-crown NPL + graphene/SiC hybrid	0.3	-0.1	-4.7	2×10^{12}	2×10^{12}

Table S1. Graphene channel: estimation of the Fermi level and carrier density absolute values and relative change for a given scenario. The relative values is the absolute value after application of a given scenario (highlighted in bold) minus the absolute value before the application of the scenario.

5. *Determination of the nanoplatelet band parameters*

In order to estimate the band alignment of the nanoplatelets *versus* graphene, we performed estimation of their electron affinity:

$$\chi_{NPL} = \chi_{Bulk} - E_C$$

where χ_{Bulk} is the electron affinity of the bulk material and E_C the confinement energy:

$$E_C = \frac{m_v{}^*}{m_v{}^* + m_c{}^*}(E_{NPL} - E_{Bulk})$$

where $m_v{}^*$ and $m_c{}^*$ are the relative mass of the hole and electron respectively, E_{NPL} the bandgap energy of the NPL and E_{Bulk} that of the bulk (Table S2).

Parameter	Bulk CdSe	Bulk CdTe
χ_{NPL}	4.95	4.28
E_{Bulk}	1.68	1.5
$m_v{}^*$	0.9	0.49
$m_c{}^*$	0.13	0.088

Table S2. Bulk material parameters from reference 10**.**

The Table S3 sums the calculated position of the conduction band (χ_{NPL}) and valence band ($\Phi_{NPL} = \chi_{NPL} + E_{NPL}$).

	CdSe core NPL	CdSe/CdS core/shell NPL	CdSe of CdSe/CdTe core/crown NPL	CdTe of CdSe/CdTe core/crown NPL
E_{NPL}	2.3	2.0	2.3	2.1
χ_{NPL}	4.4	4.6	4.4	3.8
Φ_{NPL}	6.7	6.6	6.7	5.9

Table S3. Nanoplatelets band parameters. The core and the crown have been considered as two objects since they spectroscopically behave as such.

6. Determination of exciton binding energy

Wavefunction calculation

We solve the 1D time independent Schrodinger equation

$$\left[-\frac{\hbar^2}{m^*}\frac{d^2}{dz^2}+V(z)\right]\psi(z)=E\psi(z)$$

with $\hbar$ the reduced Planck constant, $V(z)$ the 1D energy profile evaluated using the previous section, $\psi(z)$ the unknown wave function and *E* its energy, using a shooting method.[11] The equation is spatially discretized with a 0.1 nm step, so that the second derivate of the wave function can be written

$$\frac{d^2\psi}{dz^2}=\frac{\psi(z+dz)-2\psi+\psi(z-dz)}{dz^2}$$

Assuming a constant effective mass we can estimate step by step the wave function according to the equation

$$\psi(z+dz)=\left[\frac{2m^*}{\hbar^2}dz^2(V(z)-E)+2)\right]\psi(z)-\psi(z-dz)$$

We use the following spatial condition

$$\psi(0)=0 \text{ and } \psi(1)=1$$

and the energy is chosen to minimize the value of ψ_∞. We perform this equation solvation for bot electron and hole energy profile and use the obtained wavefunction to evaluate their overlap

$$S=\left|\int\psi_e(z)\psi_h^*(z)dz\right|^2$$

Exciton binding energy calculation

Knowing the exciton radiative lifetime τ and the overlap integral S between the hole and the electron wavefunction inside the nanoplatelet, the exciton binding energy can be estimated by

$$E_{exc}=\frac{2\pi\varepsilon_0 m_0 c^3\hbar^2}{ne^2E_p\tau S^2}$$

with ε_0 the vacuum permittivity, m_0 the rest mass of the electron, $\hbar$ the reduced Planck's constant, n the optical index of the medium, E_P the Kane energy ($E_P=16.5$ eV for oleic acid coated CdSe nanoplatelets,[1]) and n the refractive index of the surrounding medium (toluene, at 510 nm $n=1.5$). The

ratio of the CdSe/CdS core/shell NPL exciton binding energy over the one of CdSe core NPL thus equals to

$$\frac{E_{Exc,Core/Shell}}{E_{Exc,Core}} = \frac{(\tau S^2)_{Core}}{(\tau S^2)_{Core/Shell}}$$

Given the calculated overlap integral and measured lifetime (Table S4),

$$\frac{E_{Exc,Core/Shell}}{E_{Exc,Core}} = 0.45$$

The NPL exciton binding energy is thus divided by 2 upon the growth of a CdS shell. Knowing that $E_{Exc,Core} = 0.25$ eV,[12,13] $E_{Exc,Core/Shell} = 0.11$ eV.

	core	core/shell
Lifetime (ns)	1.1	3.0
Overlap integral S	1.0	0.9
E_{Ex} (eV)	.25	.11

Table S4. Parameters and results for exciton binding energy calculations.

7. *Performances of CdSe/CdS NPL decorated graphene photodetector*

Bare graphene does not exhibit significant photoresponse under increasing illumination, see Figure S4a.

Voltage noise spectral density under increasing bridge excitation voltage has been measured for a CdSe/CdS core/shell NPL decorated graphene channels and the results are presented in Figure S4b. A 3 parameters fit according to the equation $S_{V^2} = S_{V,Thermal}{}^2 + \frac{\beta}{f^\gamma}$ has been performed for each curve. The obtained thermal noise $S_{V,Thermal} = 24\, nV/\sqrt{Hz}$ is in good agreement with the average resistance of the channels $R = 19k\Omega$. The value of the power exponent $is\ \gamma = 1.1 \pm 0.1$ as expected for $1/f$ noise without generation-recombination bulges.

Since the specific detectivity expresses as $D^* = \frac{R\sqrt{A}}{S_n}$ where R is the responsivity, A the optical area and S_n the noise spectral density, a measurement of the responsivity under different illumination modulation frequency is necessary. It is performed by illuminating a single channel of the Wheatstone bridge and by measuring the evolution of the voltage imbalance amplitude V_O as a function of the light chopping frequency. Results are presented in Figure S4c. At a given modulation frequency, the voltage imbalance increases linearly *versus* optical power until 10 mW.cm^{-2}. At higher illumination, the signal saturates. A 3 parameters fit according to the empirical equation

$$V_O = V_\infty \frac{\left. {P_{Opt}} \middle/ {P_{Opt,sat}} \right.^{a}}{1 + \left. {P_{Opt}} \middle/ {P_{Opt,sat}} \right.}$$

has been performed in order to extract the slope V_∞ at low optical power and the saturation optical power $P_{Opt,sat}$, the exponent *a* being ≈ 1. The slope is directly related to the voltage responsivity

$$R_V = \frac{V_\infty}{P_{Opt,sat}{}^a A}$$

and is plotted in Figure S4b – inset right. Since the current responsivity can be related to the voltage responsivity by

$$R_I = \frac{2}{R_{channel}} R_V$$

and the current noise spectral density to the voltage noise density by

$$S_I = \frac{1}{2R_{channel}} S_V$$

where $R_{channel}$ is the resistance of a single graphene channel, the specific detectivity can be expressed as

$$D^* = \frac{4V_\infty}{P_{Opt}\sqrt{A}S_V}.$$

We eventually checked the spectral photoresponse of the device by measuring the change in conductance relative to dark conditions upon illumination at different wavelengths (see Figure S4d black curve, the large errors bars are due to fact that the photodetector response is slower than the wavelength sweep speed from 500 nm to 800 nm). The photoresponse spectrum agrees well to the absorbance spectrum of a CdSe/CdS NPL suspension (Figure S4d, red curve).

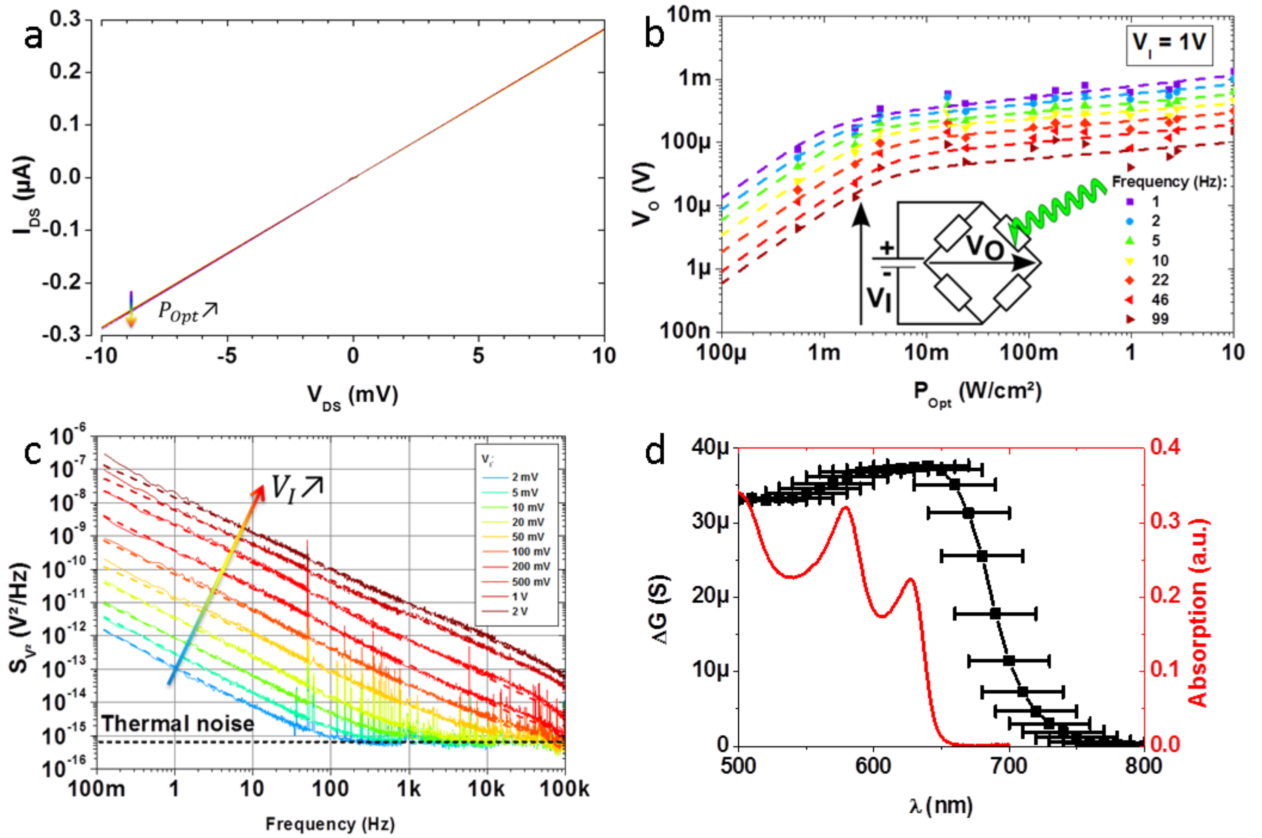

Figure S4. (a) Current *versus* voltage of a bare graphene channel under increasing illumination. (b) Voltage imbalance *versus* illumination power at different chopping frequencies for a CdSe/CdS NPL decorated graphene photodetector. The measurements are performed under a 1V bridge excitation voltage. Dashed-line: 3 parameters fit $V_O = V_\infty \frac{\left(P_{Opt}/P_{Opt,sat}\right)^a}{1+P_{Opt}/P_{Opt,sat}}$. Inset: scheme of the device where a single channel is illuminated. (c) Square voltage noise spectral densities of a CdSe/CdS core/shell NPL decorated graphene channel under different Wheatstone bridge excitation voltages V_I. Dashed line: fit according to equation $S_{V^2} = S_{V,Thermal}{}^2 + \frac{\beta}{f^\gamma}$. (d) Conductance change at different illumination wavelengths of a CdSe/CdS NPL decorated graphene photodetector (black), absorption spectrum of a suspension of CdSe/CdS NPL (red).

8. *Effect of the nanoplatelets film thickness on the photoconduction properties*

We investigate the effect of the film thickness on the phototransistor conductance properties. We evidence from Figure 2a that no photoresponse is observed in absence of NPL. On the other hand, in the thick film limit, we observe no dependence of the thickness on the charge transfer (Figure S5). As a result we believe that most of the charge transfer result from the very first layer of NPL at the graphene interface.

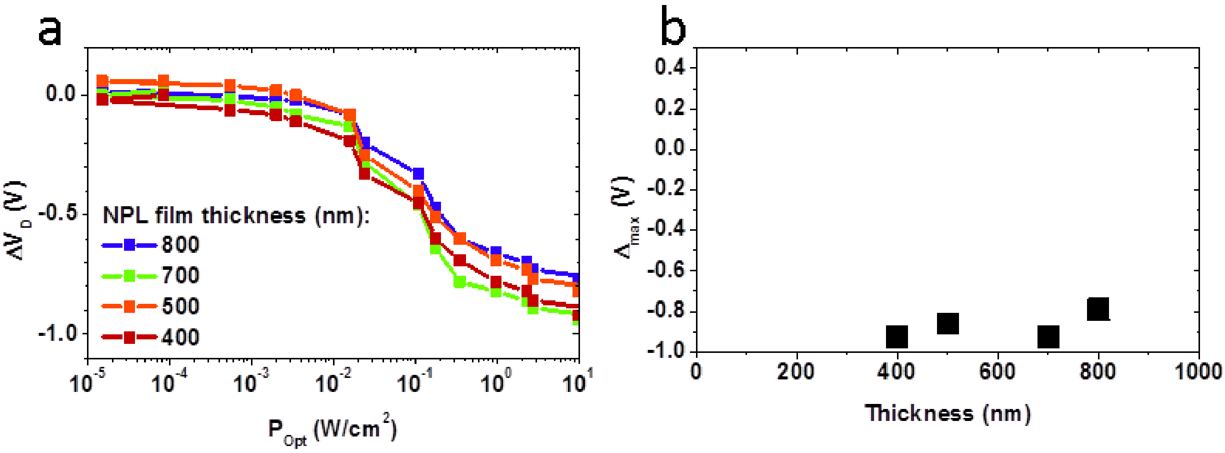

Figure S5. (a) Evolution of the maximal Dirac point voltage displacement versus CdSe/CdS core/shell NPL film thickness. (b) Displacement of the Dirac point voltage versus incident optical power for CdSe/CdS core/shell NPL films of different thicknesses.

9. References

1. Ithurria, S. *et al.* Colloidal nanoplatelets with two-dimensional electronic structure. *Nat. Mater.* **10,** 936–941 (2011).
2. Nag, A. *et al.* Metal-free Inorganic Ligands for Colloidal Nanocrystals: S2–, HS–, Se2–, HSe–, Te2–, HTe–, TeS32–, OH–, and NH2– as Surface Ligands. *J. Am. Chem. Soc.* **133,** 10612–10620 (2011).
3. Ithurria, S. & Talapin, D. V. Colloidal Atomic Layer Deposition (c-ALD) using Self-Limiting Reactions at Nanocrystal Surface Coupled to Phase Transfer between Polar and Nonpolar Media. *J. Am. Chem. Soc.* **134,** 18585–18590 (2012).
4. Pedetti, S., Ithurria, S., Heuclin, H., Patriarche, G. & Dubertret, B. Type-II CdSe/CdTe Core/Crown Semiconductor Nanoplatelets. *J. Am. Chem. Soc.* **136,** 16430–16438 (2014).
5. Pallecchi, E. *et al.* High Electron Mobility in Epitaxial Graphene on 4H-SiC(0001) via post-growth annealing under hydrogen. *Sci. Rep.* **4,** 4558 (2014).
6. Lhuillier, E. *et al.* Electrolyte-Gated Field Effect Transistor to Probe the Surface Defects and Morphology in Films of Thick CdSe Colloidal Nanoplatelets. *ACS Nano* **8,** 3813–3820 (2014).
7. Froehlicher, G. & Berciaud, S. Raman spectroscopy of electrochemically gated graphene transistors: Geometrical capacitance, electron-phonon, electron-electron, and electron-defect scattering. *Phys. Rev. B* **91,** 205413 (2015).
8. Lhuillier, E., Robin, A., Ithurria, S., Aubin, H. & Dubertret, B. Electrolyte-Gated Colloidal Nanoplatelets-Based Phototransistor and Its Use for Bicolor Detection. *Nano Lett.* **14,** 2715–2719 (2014).
9. Das, A. *et al.* Monitoring dopants by Raman scattering in an electrochemically top-gated graphene transistor. *Nat. Nanotechnol.* **3,** 210–215 (2008).
10. Swank, R. K. Surface Properties of II-VI Compounds. *Phys. Rev.* **153,** 844–849 (1967).
11. Harrison, P. *Quantum Wells, Wires and Dots: Theoretical and Computational Physics of Semiconductor Nanostructures*. (John Wiley & Sons, 2005).
12. Benchamekh, R. *et al.* Tight-binding calculations of image-charge effects in colloidal nanoscale platelets of CdSe. *Phys. Rev. B* **89,** 035307 (2014).
13. Achtstein, A. W. *et al.* Electronic Structure and Exciton–Phonon Interaction in Two-Dimensional Colloidal CdSe Nanosheets. *Nano Lett.* **12,** 3151–3157 (2012).
